# Multi-Target Stool DNA Testing for Colorectal Cancer Screening: Emerging Learning on Real-world Performance

**DOI:** 10.1007/s11938-020-00271-5

**Published:** 2020-01-21

**Authors:** Jason D. Eckmann, Derek W. Ebner, John B. Kisiel

**Affiliations:** 1 Department of Internal Medicine, Mayo Clinic, Rochester, MN, USA; 2 Division of Gastroenterology and Hepatology, Mayo Clinic, 200 First Street SW, Rochester, MN, 55902, USA

**Keywords:** Colorectal neoplasms/prevention and control, Colorectal neoplasms/diagnosis, DNA, neoplasm/analysis, Early detection of cancer/methods, Colonoscopy/trends, Proximal colorectal neoplasia, Pre-cancerous conditions/diagnosis

## Abstract

**Purpose of Review:**

Multi-target stool DNA (MT-sDNA) was approved in 2014 for use in screening average-risk patients for colorectal cancer (CRC). Here, we highlight recent literature from post-market studies to provide an update on clinical use and utility not possible from pre-approval studies.

**Recent Findings:**

MT-sDNA has been included in major society guidelines as an option for colorectal cancer screening, and has seen exponentially increasing use in clinical practice. MT-sDNA appears to be attracting new patients to CRC screening, and patient adherence to diagnostic colonoscopy after a positive MT-sDNA test is high. Approximately two-thirds of these patients are found to have colorectal neoplasia (CRN), 80% of whom have at least one right-sided lesion; 1 in 3 will have advanced CRN. High yield of CRN is due not only to post-screening increase in probability but also likely improved endoscopist attention. In those with a negative high-quality colonoscopy after positive MT-sDNA test (“false positive MT-sDNA”), further interventions do not appear to be necessary.

**Summary:**

MT-sDNA is a promising tool to improve rates and quality of CRC screening. Further investigation should examine MT-sDNA performance in populations at increased risk for CRC, and as an interval test after colonoscopy to detect potentially missed lesions.

Colorectal cancer (CRC) is a major cause of cancer-related death in the USA [1], with approximately 150,000 new diagnoses and 50,000 deaths from CRC projected in 2019 alone [2]. With widespread screening initiatives to detect CRC and pre-cancerous polyps, CRC-related morbidity and mortality have decreased in recent years [2]. The US Preventive Services Task Force (USPSTF) recommends screening for CRC in average-risk adults between the ages of 50 and 75 [3], while more recent recommendations from the American Cancer Society (ACS) suggest initiating screening at age 45 in light of the increasing rate of CRC diagnosed in younger patients [4•,^5^,^6^,^7^,^8^]. However, despite these guidelines and extensive public health initiatives, approximately one-third of screen-eligible adults in the USA remain unscreened [9, 10•].

Several methods are recommended for CRC screening [3, 4•, 11•]. Colonoscopy is the most widely used modality in the USA [9] and permits simultaneous diagnostic and therapeutic intervention for colorectal neoplasia (CRN). However, screening colonoscopy has several notable drawbacks, including reduced sensitivity in the proximal colon [12–18•] and inter-operator variability in colonoscopy quality [19–22]. Furthermore, its invasive nature; risk of complications; and the inconvenience of preparation, sedation, and time away from work are recognized barriers to patients considering screening [23]. Several non-invasive screening modalities are endorsed as an alternative to colonoscopy for use in average-risk patients. Guaiac-based fecal occult blood testing (gFOBT) has the strongest body of evidence demonstrating a reduction in CRC-related mortality [24–27]. However, dietary and medication interactions, poor performance in the proximal colon, and issues with adherence to the recommended yearly screening interval have led to decreased utilization of this test [28–30]. The introduction of fecal immunochemical testing (FIT) addressed some of these limitations, with FIT included as a first-line option in major society screening guidelines [3, 11•]. A recent meta-analysis suggested moderate-to-high sensitivity and specificity for CRC and advanced adenoma (AA) in a singleuse setting, but performance varied by assay and positivity cutoff [31]. Concerns remain regarding reduced sensitivity for right-sided and sessile serrated precursor lesions [28, 29, 32•] in addition to persistent poor adherence to required annual testing [30, 33–36].

## Multi-target stool DNA testing

Multi-target stool DNA (MT-sDNA) is a newer stool-based screening modality, with one commercial product available to patients in the USA (Cologuard^™^, Exact Sciences, Madison, WI). This test detects abnormal DNA markers (aberrantly methylated *BMP3* and *NDRG4*, mutant *KRAS*) shed from neoplastic cells in the gastrointestinal tract. Another marker, *β-actin*, serves as a control to ensure adequate quantity of DNA in the sample, while an immunochemical assay for hemoglobin detects evidence of gastrointestinal bleeding. These components are measured quantitatively and incorporated into a logistic regression algorithm containing mathematical interaction variables for each marker. These interaction terms result in numerous marker combinations that can trigger a pre-specified threshold score, resulting in a positive or negative test result that is more than the sum of its individual components [37].

MT-sDNA testing is performed by the patients in the comfort of their own home. Following a provider order, a manufacturer-based patient navigation office contacts the patient and arranges sample collection via a courier-delivered kit [38]. This system also provides patient education and test completion reminders which have been shown to result in adherence to testing of over 70% in Medicare beneficiaries [38]. Following delivery of the MT-sDNA kit, patients collect a whole stool sample, swab a portion to collect the FIT sample, add a preservative buffer to the whole stool, seal the container, and mail the samples in one box back to the laboratory for analysis.

MT-sDNA testing was approved for screening in average-risk adults by the US Food and Drug Administration (FDA) in 2014, following a large screen–setting trial by Imperiale et al. (DeeP-C trial), comparing MT-sDNA and FIT. Overall, the sensitivity of MT-sDNA for detecting CRC in this study was 92% (vs 74% for FIT, *p* = 0.002), and 42% for advanced pre-cancerous lesions (vs 24% for FIT, *p* < 0.001) [39]. A second study performed in an Alaska Native cohort confirmed these findings, and redemonstrated increasing sensitivity by adenoma size, with 80% sensitivity for lesions > 3 cm, and 100% sensitivity for CRC [40••]. Specificity was 87–94% across these two studies, and increased in younger patients (Table 1) [39, 40••]. MT-sDNA appears to have similar performance for the detection of both conventional adenomas and sessile serrated polyps (SSPs). Large SSPs and hyperplastic polyps (≥ 1 cm) were included in the sensitivity estimate for advanced pre-cancerous lesions in the DeeP-C study [39]. For greater clarity, a post hoc analysis of DeeP-C data was performed on all lesions ≥ 1 cm with histology confirmed for SSP. The median size was 1.2 cm (range 1–3), and the majority (28/29 (93%)) were located proximal to the splenic flexure; only one SSP contained dysplasia. In this setting, MT-sDNA had a sensitivity of 55% (95% CI, 36–74%) and a specificity of 91% [41]. MT-sDNA performance characteristics for the individual endpoint category of large hyperplastic polyp have not been estimated and traditional serrated adenomas were not identified by screening colonoscopy in the DeeP-C study; these endpoints are deserving of future study.

Population modeling studies performed as part of a USPSTF review suggest that programmatic use of MT-sDNA would result in fewer lifetime colonoscopies than FIT and a stronger benefit-to-harms ratio than colonoscopy (Table 2) [3]. As a result of the above findings, MT-sDNA testing was subsequently endorsed by the USPSTF in their 2016 guidelines for CRC screening [3]. Utilization of MT-sDNA has been increasing exponentially since then, with nearly 3 million screens performed to date [42].

### Outcomes in patients undergoing multi-target stool DNA testing

Since the introduction of MT-sDNA in 2014, data have begun to emerge regarding its “real-world” performance. MT-sDNA appears to be attracting new patients to screening. One recent study demonstrated high utilization of MT-sDNA in almost 400 previously non-adherent average-risk Medicare patients, with 51% harboring advanced neoplastic lesions; fewer than 20% had no neoplastic findings at diagnostic colonoscopy [43•]. Furthermore, we recently presented data from a cohort of over 2000 MT-sDNA-positive patients showing that approximately 25% had never been screened for CRC and were > 10 years overdue [44]. While there may be many reasons for patients to go without screening, these findings suggest that MT-sDNA is attracting previously nonadherent patients, a critical outcome in the ongoing long-term fight to reduce CRC-attributable mortality.

Of all patients screened with MT-sDNA, 14–16% will have positive results [39, 44, 45]. Following a positive MT-sDNA test, it is imperative that patients undergo diagnostic colonoscopy to evaluate for CRN. While previous estimates of adherence to diagnostic colonoscopy ranged from 75 to 96% [43•, 45], our recent analysis showed a diagnostic colonoscopy completion rate of ~ 90% in a large multi-practice setting [44]. Reasons for non-adherence to recommended diagnostic colonoscopy vary, but are commonly related to a wish to avoid colonoscopy, in addition to medical comorbidities that preclude sedation and procedural intervention [45]. These factors illustrate the importance of both provider and patient education prior to pursuing MT-sDNA testing in order to ensure selection of appropriate patients for screening with this test.

In patients with positive MT-sDNA testing who undergo diagnostic colonoscopy, our data show that approximately two-thirds will be found to have at least one neoplastic lesion [44]. Among patients with CRN, 40% will have at least one advanced lesion (defined as CRC, adenomas, or SSPs with at least one of the following characteristics: ≥ 1 cm in size, high-grade dysplasia, or having ≥ 25% villous elements), and ~ 1% will have CRC. SSPs will be detected in about half of patients with positive tests.

Importantly, MT-sDNA testing appears to perform well in detecting rightsided (proximal) neoplasia. In our multi-site cohort of patients with positive MT-sDNA tests, over 50% of patients overall (and 80% of those with neoplastic lesions) were found to have proximal CRN [44]. The reason for the high yield of proximal CRC is likely multifactorial, although improved detection resulting from the multiple markers in MT-sDNA offers one potential explanation. Right-sided lesions tend to be flat and less likely to bleed [46], and exfoliation of DNA markers is more consistent throughout the colon [47]; therefore, MT-sDNA has an advantage over other non-invasive methods for the detection of right-sided neoplasia.

**Fig. 1.**
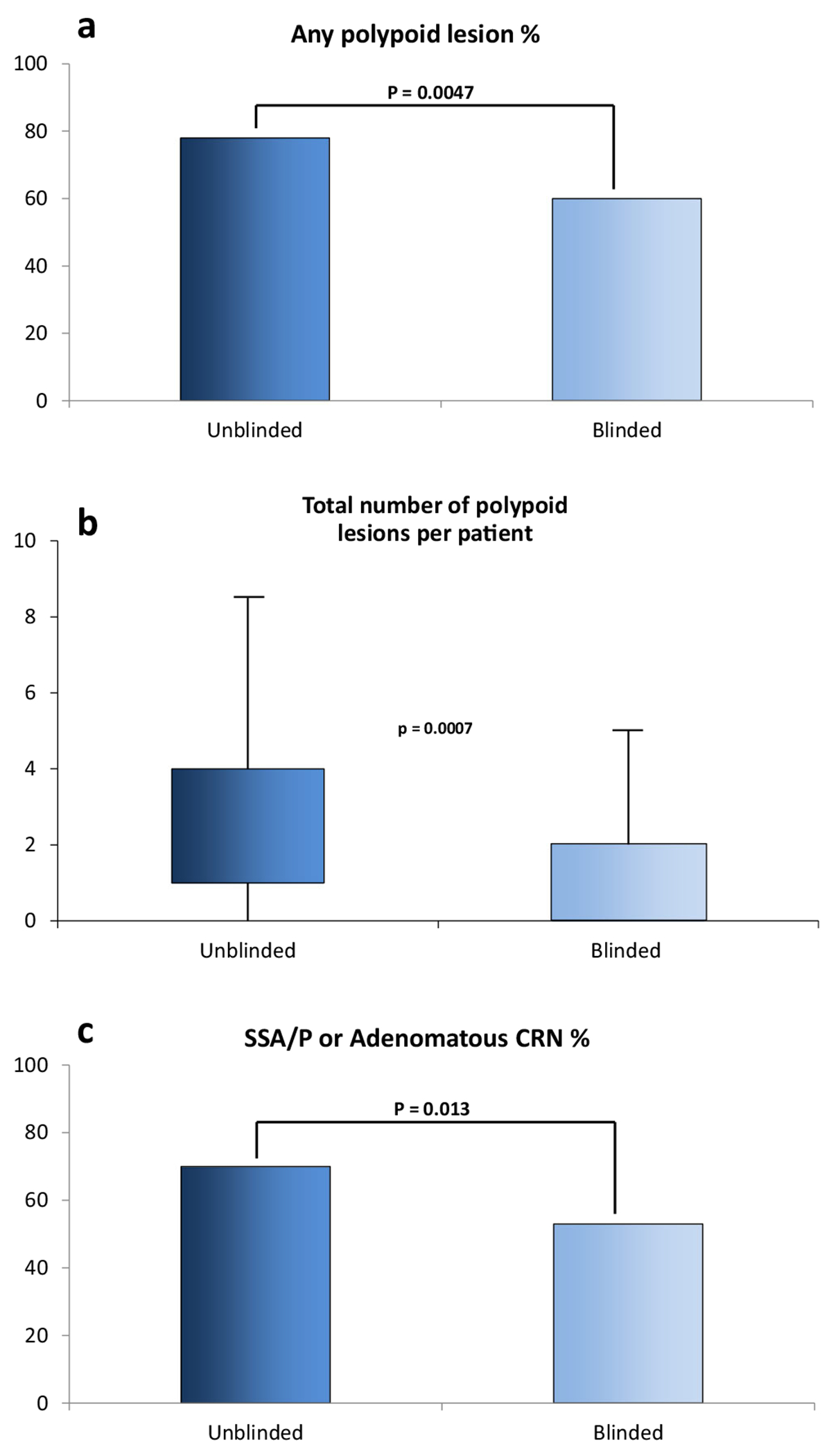
The **a** proportion of patients with polyps, **b** number of polyps per patient, and **c** proportion of patients with sessile serrated and adenomatous polyps found at diagnostic colonoscopy after positive multi-target stool DNA test is higher in unblinded endoscopists compared with blinded endoscopists. Used with permission from Elsevier.

These point estimates for positive predictive value appear higher than reported in the pre-FDA approval trial, where advanced lesions were found in 24% of those with positive MT-sDNA tests [39]. This is likely in part due to underlying differences in the populations being studied, or in differences in those opting to use the test in clinical practice versus the clinical trial setting. However, there are several other factors that likely contribute to the high yield of MT-sDNA for CRN, both proximal and distal. First, by screening an average-risk population with MT-sDNA and performing further testing only in those with positive results, this creates an enriched population in whom the pre-test probability of CRN at diagnostic colonoscopy is increased. Furthermore, studies have shown that endoscopist knowledge of a positive MT-sDNA test significantly increases quality metrics such as ADR at diagnostic colonoscopy compared with blinded endoscopists, likely related to longer withdrawal time and increased attention to subtle lesions (Fig. 1) [48••]. Moreover, recent data suggest that while improvement in quality and yield of diagnostic colonoscopy is observed in all endoscopists in a large academic practice, the greatest improvement in polyp detection is seen in gastroenterologists with lower baseline detection rates [49].

### “False positive” MT-sDNA tests

A common question among physicians and other health care providers is what should be done in the setting of a positive MT-sDNA test followed by a negative colonoscopy (a “false positive” MT-sDNA test). However, before addressing this question, negative colonoscopy needs to be more clearly defined. The original studies investigating MT-sDNA testing labeled true positive tests as those in which only advanced neoplasia or CRC was detected [39]. However, we have shown that approximately 40% of lesions detected by MT-sDNA are non-advanced [44]; a positive MT-sDNA test in these situations is therefore unlikely to be biologically false, but rather to have detected exfoliated DNA from a neoplasm not meeting arbitrary size-based criteria. With this in mind, knowing that 14% of all MT-sDNA tests are positive and that 67% of these patients have CRN found at diagnostic colonoscopy, less than 5% of all patients screened with MT-sDNA would have no polyps found at colonoscopy (14.1% ϗ (100% – 67%) = 4.7%). Even when including only advanced CRN and CRC, 10% (14.1% χ (100% – 28%)) would have false positive results.

In the clinical situation of a false positive MT-sDNA test, the next step is to ensure that a diagnostic colonoscopy of sufficient quality was performed. In clinical trials of MT-sDNA, only those with high-quality colonoscopy, defined as having good or excellent bowel preparation, photographic evidence of cecal intubation, and withdrawal time of ≥ 6 min, were included in primary study outcomes [21, 39, 40••, 50]. Assuming a high-quality colonoscopy, current guidelines suggest that no further testing be performed [11•]. These recommendations are based on several studies that found no increased risk of CRC in this specific population. One such study prospectively evaluated 30 patients with initial false positive MT-sDNA testing (evaluated by both colonoscopy and esophagogastroduodenoscopy (EGD)), who underwent repeat MT-sDNA and colonoscopy/EGD 1 year later. In this cohort, only two advanced lesions were found on repeat testing; however, information on initial colonoscopy quality was not reported [51•]. Another study retrospectively analyzed approximately 1000 patients with either false positive or true negative MT-sDNA tests, and found no increased rate of aerodigestive cancers in a per-protocol analysis at a median follow-up of 4 years [52•]. More recently, a multi-site retrospective cohort study performed by Berger et al. evaluated approximately 1200 patients with negative colonoscopy over a median of 5 years, and stratified patients by concordant (negative) or discordant (positive) MT-sDNA results. They found no increased incidence of aerodigestive cancers in either group, with rates that were equivalent to the general population based on Surveillance, Epidemiology, and End Results (SEER) Program data [53•]. Therefore, based on currently available evidence, clinicians should not recommend repeat colonoscopy, EGD, or other further testing in patients with positive MT-sDNA, a negative high-quality diagnostic colonoscopy, and no localizing signs or symptoms that would mandate evaluation.

### Future directions

Even with increasing utilization of MT-sDNA and the emergence of postapproval performance data, several opportunities for further study remain. While the current recommended testing interval for MT-sDNA is 3 years, this is based on manufacturer’s recommendations and computer modeling, and further investigation is needed into MT-sDNA performance with different testing frequencies. Additional study of patient adherence to both the testing schedule and diagnostic colonoscopy in other populations would also help to further inform test performance in the real world.

MT-sDNA testing may benefit additional patient populations. In response to the ACS recommendation to begin screening for CRC at age 45, studies conducted in archival tissue specimens demonstrated that MT-sDNA markers in primary CRC tumors among those 45–49 years old at diagnosis are similar to those seen in tumors of patients aged 50–64 [54]; methylated DNA markers were all substantially higher than observed in control tissues across these age ranges. As a result of these data and those expected from a clinical trial utilizing MT-sDNA in patients age 45–49 (ClinicalTrials.gov Identifier: NCT03728348), the FDA recently expanded the label for MT-sDNA to include those age 45–84.

The FDA’s current labeling excludes those at increased risk for CRC due to conditions such as inflammatory bowel disease (IBD), personal history of advanced CRN, family history of early CRC, and signs of blood loss, among others. Initial data from our group’s evaluation of off-label use of MT-sDNA testing (~ 10% of tests) in patients with these risk factors show that test performance is similar to those at average risk [55]. Furthermore, in a research study of 192 patients with IBD undergoing MT-sDNA testing for surveillance, the test demonstrated similar sensitivity for both CRC and CRN when compared with the general population [56•]. However, further study is needed before extending MT-sDNA use into these increased-risk populations.

Finally, MT-sDNA may have important utility as an interval test following apparently negative colonoscopy. In our group’s analysis of data in patients undergoing MT-sDNA testing within 5 years of a negative colonoscopy, the yield of all CRN and most CRN subtypes was the same as in those undergoing testing 10 or more years after colonoscopy. When these patients were further stratified by whether their prior colonoscopy was complete or incomplete (e.g., poor prep, no cecal intubation), neoplastic yield remained similar between groups [57]. Advanced neoplasms were found in 16–17% of those screened early with MT-sDNA; these findings could not be explained by known elevations in baseline risk. Taken together, these findings suggest that lesions are likely being missed at screening colonoscopies. MT-sDNA should therefore be studied as a tool between colonoscopies to identify those in which polyps were likely to have been missed, and thereby reduce the risk of interval CRC.

## Conclusion

MT-sDNA is a stool-based screening test with high sensitivity and specificity for both CRN and CRC. As its use has increased over the past 5 years after FDA approval, it has attracted a large population of previously non-adherent patients to screening. A majority of patients with a positive test are found to have CRN at diagnostic colonoscopy, with most harboring right-sided lesions. While further investigation is necessary into additional potential uses, MT-sDNA has firmly cemented itself as a convenient, non-invasive, and effective tool for widespread screening in the ongoing fight against CRC.

## Figures and Tables

**Table 1.** Multi-target stool DNA demonstrates higher specificity in younger patients with normal colonoscopy

Patient Population	Specificity
Imperiale et al [39]	
No CRC or advanced adenoma*, all ages	86.6%
Negative colonoscopy, all ages	90%
No CRC or advanced adenoma*, age 50-64	91.5%
Negative colonoscopy, age 50-64	94%
Redwood et al [40]	
No CRC or advanced adenoma*, all ages	91%
Negative colonoscopy, all ages	93%

* Patients with non-advanced lesions, hyperplastic polyps and other findings (e.g. diverticulitis) included in specificity calculation

**Table 2.** Screening with multi-target stool DNA testing results in fewer lifetime colonoscopies than FIT, and has a superior benefit-to-harm ratio compared to colonoscopy

Screening Modality	Life years gained*	CRC deaths averted*	Complications (GI/CV)*	Colonoscopies*	Life years gained/complication	Life years gained/colonoscopy	CRC deaths averted/complication	CRC deaths averted colonoscopy
MT-sDNA	226	20	9	1714	25	0.13	2.2	0.01
FIT	244	22	10	1757	24	0.14	2.2	0.01
Colonoscopy	270	24	15	4049	18	0.07	1.6	0.006

Adapted from: Bibbens-Domingo, et al. [3]

* Per 1000 persons screened

## References

[1] Cronin KA, Lake AJ, Scott S, Sherman RL, Noone AM, Howlader N, Annual Report to the Nation on the Status of Cancer, part I: national cancer statistics. Cancer. 2018;124(13):2785–800. 10.1002/cncr.31551. 29786848 PMC6033186

[2] Siegel RL, Miller KD, Jemal A. Cancer statistics, 2019. CA Cancer J Clin. 2019;69(1):7–34. 10.3322/caac.21551. 30620402

[3] Bibbins-Domingo K, Grossman DC, Curry SJ, Davidson KW, Epling JW Jr, Garcia FAR, Screening for colorectal cancer: US Preventive Services Task Force recommendation statement. JAMA. 2016;315(23):2564–75. 10.1001/jama.2016.5989. 27304597

[4.•] AMD W, ETH F, Church TR, Flowers CR, Guerra CE, SJ LM, Colorectal cancer screening for average-risk adults: 2018 guideline update from the American Cancer Society. CA Cancer J Clin. 2018;68(4):250–81. 10.3322/caac.21457. 29846947

[5] Ward EM, Sherman RL, Henley SJ, Jemal A, Siegel DA, Feuer EJ, Annual Report to the Nation on the Status of Cancer, featuring cancer in men and women age 20–49 years. JNCI: Journal of the National Cancer Institute. 2019. 10.1093/jnci/djz106. PMC6910179 31145458

[6] Bailey CE, Hu CY, You YN, Bednarski BK, Rodriguez-Bigas MA, Skibber JM, Increasing disparities in the age-related incidences of colon and rectal cancers in the United States, 1975-2010. JAMA surgery. 2015;150(1):17–22. 10.1001/jamasurg.2014.1756. 25372703 PMC4666003

[7] Hessami Arani S, Kerachian MA. Rising rates of colorectal cancer among younger Iranians: is diet to blame? Curr Oncol. 2017;24(2):e131–e7. 10.3747/co.23.3226. 28490936 PMC5407876

[8] Troeung L, Sodhi-Berry N, Martini A, Malacova E, Ee H, O’Leary P, Increasing incidence of colorectal cancer in adolescents and young adults aged 15-39 years in Western Australia 1982-2007: examination of colonoscopy history. Front Public Health. 2017;5:179. 10.3389/fpubh.2017.00179. 28791283 PMC5522835

[9] Control CD. Vital signs: colorectal cancer screening test use–United States, 2012. MMWR Morb Mortal Wkly Rep. 2013;62(44):881–8. 24196665 PMC4585592

[10.•] Joseph DA, King JB, Richards TB, Thomas CC, Richardson LC. Use of colorectal cancer screening tests by state. Prev Chronic Dis. 2018;15:E80. 10.5888/pcd15.170535. 29908051 PMC6016405

[11.•] Rex DK, Boland RC, Dominitz JA, Giardiello FM, Johnson DA, Kaltenbach T Colorectal cancer screening: recommendations for physicians and patients from the U.S. Multi-Society Task Force on colorectal cancer. Am J Gastroenterol. 2017;112(7):1016–1030. doi:10.1038/ajg.2017.174. 28555630

[12] Nishihara R, Wu K, Lochhead P, Morikawa T, Liao X, Qian ZR, Long-term colorectal-cancer incidence and mortality after lower endoscopy. N Engl J Med. 2013;369(12):1095–105. 10.1056/NEJMoa1301969. 24047059 PMC3840160

[13] Xiang L, Zhan Q, Zhao XH, Wang YD, An SL, Xu YZ, Risk factors associated with missed colorectal flat adenoma: a multicenter retrospective tandem colonoscopy study. World J Gastroenterol. 2014;20(31):10927–37. 10.3748/wjg.v20.i31.10927. 25152596 PMC4138473

[14] Singh H, Nugent Z, Demers AA, Kliewer EV, Mahmud SM, Bernstein CN. The reduction in colorectal cancer mortality after colonoscopy varies by site of the cancer. Gastroenterology. 2010;139(4):1128–37. 10.1053/j.gastro.2010.06.052. 20600026

[15] Kahi CJ, Hewett DG, Norton DL, Eckert GJ, Rex DK. Prevalence and variable detection of proximal colon serrated polyps during screening colonoscopy. Clin Gastroenterol Hepatol. 2011;9(1):42–6. 10.1016/j.cgh.2010.09.013. 20888435

[16] Baxter NN, Goldwasser MA, Paszat LF, Saskin R, Urbach DR, Rabeneck L. Association of colonoscopy and death from colorectal cancer. Ann Intern Med. 2009;150(1):1–8. 19075198 10.7326/0003-4819-150-1-200901060-00306

[17] Brenner H, Hoffmeister M, Arndt V, Stegmaier C, Altenhofen L, Haug U. Protection from right-and leftsided colorectal neoplasms after colonoscopy: population-based study. J Natl Cancer Inst. 2010;102(2):89–95. 10.1093/jnci/djp436. 20042716

[18.•] Lee JK, Jensen CD, Levin TR, Zauber AG, Schottinger JE, Quinn VP, Long-termrisk of colorectal cancer and related deaths after a colonoscopy with normal findings. JAMA Intern Med. 2018. 10.1001/jamainternmed.2018.5565. PMC6439662 30556824

[19] Corley DA, Levin TR, Doubeni CA. Adenoma detection rate and risk of colorectal cancer and death. N Engl J Med. 2014;370(26):2541. 10.1056/NEJMc1405329. 24963577

[20] Butterly L, Robinson CM, Anderson JC, Weiss JE, Goodrich M, Onega TL, Serrated and adenomatous polyp detection increases with longer withdrawal time: results from the New Hampshire Colonoscopy Registry. Am J Gastroenterol. 2014;109(3):417–26. 10.1038/ajg.2013.442. 24394752 PMC4082336

[21] Barclay RL, Vicari JJ, Doughty AS, Johanson JF, Greenlaw RL. Colonoscopic withdrawal times and adenoma detection during screening colonoscopy. N Engl J Med. 2006;355(24):2533–41. 10.1056/NEJMoa055498. 17167136

[22] Rex DK, Schoenfeld PS, Cohen J, Pike IM, Adler DG, Fennerty MB, Quality indicators for colonoscopy. Am J Gastroenterol. 2015;110(1):72–90. 10.1038/ajg.2014.385. 25448873

[23] Steinwachs D, Allen JD, Barlow WE, Duncan RP, Egede LE, Friedman LS, National Institutes of Health state-of-the-science conference statement: enhancing use and quality of colorectal cancer screening. Ann Intern Med. 2010;152(10):663–7. 10.7326/0003-4819-152-10-201005180-00237. 20388702

[24] Shaukat A, Mongin SJ, Geisser MS, Lederle FA, Bond JH, Mandel JS, Long-term mortality after screening for colorectal cancer. N Engl J Med. 2013;369(12):1106–14. 10.1056/NEJMoa1300720. 24047060

[25] Faivre J, Dancourt V, Lejeune C, Tazi MA, Lamour J, Gerard D, Reduction in colorectal cancer mortality by fecal occult blood screening in a French controlled study. Gastroenterology. 2004;126(7):1674–80. 10.1053/j.gastro.2004.02.018. 15188160

[26] Scholefield JH, Moss SM, Mangham CM, Whynes DK, Hardcastle JD. Nottingham trial of faecal occult blood testing for colorectal cancer: a 20-year follow-up. Gut. 2012;61(7):1036–40. 10.1136/gutjnl-2011-300774. 22052062

[27] Kronborg O, Fenger C, Olsen J, Jorgensen OD, Sondergaard O. Randomised study of screening for colorectal cancer with faecal-occult-blood test. Lancet. 1996;348(9040):1467–71. 10.1016/s0140-6736(96)03430-7. 8942774

[28] Haug U, Kuntz KM, Knudsen AB, Hundt S, Brenner H. Sensitivity of immunochemical faecal occult blood testing for detecting left- vs right-sided colorectal neoplasia. Br J Cancer. 2011;104(11):1779–85. 10.1038/bjc.2011.160. 21559011 PMC3111170

[29] Hirai HW, Tsoi KK, Chan JY, Wong SH, Ching JY, Wong MC, Systematic review with meta-analysis: faecal occult blood tests show lower colorectal cancer detection rates in the proximal colon in colonoscopyverified diagnostic studies. Aliment Pharmacol Ther. 2016;43(7):755–64. 10.1111/apt.13556. 26858128

[30] Fenton JJ, Elmore JG, Buist DS, Reid RJ, Tancredi DJ, Baldwin LM. Longitudinal adherence with fecal occult blood test screening in community practice. Ann Fam Med. 2010;8(5):397–401. 10.1370/afm.1133. 20843880 PMC2939414

[31] Imperiale TF, Gruber RN, Stump TE, Emmett TW, Monahan PO. Performance characteristics of fecal immunochemical tests for colorectal cancer and advanced adenomatous polyps: a systematic review and metaanalysis. Ann Intern Med. 2019. 10.7326/m18-2390. 30802902

[32.•] Zorzi M, Hassan C, Capodaglio G, Narne E, Turrin A, Baracco M, Divergent long-term detection rates of proximal and distal advanced neoplasia in fecal immunochemical test screening programs: a retrospective cohort study. Ann Intern Med. 2018;169(9):602–9. 10.7326/m18-0855. 30285055

[33] Jensen CD, Corley DA, Quinn VP, Doubeni CA, Zauber AG, Lee JK, Fecal immunochemical test program performance over 4 rounds of annual screening: a retrospective cohort study. Ann Intern Med. 2016;164(7):456–63. 10.7326/m15-0983. 26811150 PMC4973858

[34] Liang PS, Wheat CL, Abhat A, Brenner AT, Fagerlin A, Hayward RA, Thomas JP, Vijan S, Inadomi JM Adherence to competing strategies for colorectal cancer screening over 3 years. Am J Gastroenterol 2016;111(1):105–114. doi:10.1038/ajg.2015.367. 26526080 PMC4887132

[35] Gellad ZF, Stechuchak KM, Fisher DA, Olsen MK, McDuffie JR, Ostbye T, Longitudinal adherence to fecal occult blood testing impacts colorectal cancer screening quality. Am J Gastroenterol. 2011;106(6):1125–34. 10.1038/ajg.2011.11. 21304501

[36] Cyhaniuk A, Coombes ME. Longitudinal adherence to colorectal cancer screening guidelines. Am J Manag Care. 2016;22(2):105–11. 26885670

[37] Lidgard GP, Domanico MJ, Bruinsma JJ, Light J, Gagrat ZD, Oldham-Haltom RL, Clinical performance of an automated stool DNA assay for detection of colorectal neoplasia. Clin Gastroenterol Hepatol. 2013;11(10):1313–8. 10.1016/j.cgh.2013.04.023. 23639600

[38] Swartz R, Weiser E, Parks P, Van Thomme J, Limburg P, Berger BM. Su1660: Colorectal cancer screening: compliance with multitarget stool Dna testing among Medicare beneficiaries. Gastroenterology. 2019;156(6):S–601. 10.1016/S0016-5085(19)38398-2.

[39] Imperiale TF, Ransohoff DF, Itzkowitz SH. Multitarget stool DNA testing for colorectal-cancer screening. N Engl J Med. 2014;371(2):187–8. 10.1056/NEJMc1405215. 25006736

[40.••] Redwood DG, Asay ED, Blake ID, Sacco PE, Christensen CM, Sacco FD, Stool DNA testing for screening detection of colorectal neoplasia in Alaska Native people. Mayo Clin Proc. 2016;91(1):61–70. 10.1016/j.mayocp.2015.10.008. 26520415

[41] Heigh RI, Yab TC, Taylor WR, Hussain FTN, Smyrk TC, Mahoney, Detection of colorectal serrated polyps by stool DNA testing: comparison with fecal immunochemical testing for occult blood (FIT). PLoS One. 2014;9(1):e85659. 10.1371/journal.pone.0085659. 24465639 PMC3896420

[42] Connelly C. Cologuard helpsmore people get screened in a cost-effective way. 2019.

[43.•] Prince M, Lester L, Chiniwala R, Berger B. Multitarget stool DNA tests increases colorectal cancer screening among previously noncompliant Medicare patients. World J Gastroenterol. 2017;23(3):464–71. 10.3748/wjg.v23.i3.464. 28210082 PMC5291851

[44] Eckmann JD, Ebner D, Bering J, Kahn A, Rodriguez EA, Devens ME, Su1664: High yield of total and rightsided colorectal neoplasia by multi-target stool Dna testing in average risk patients irrespective of prior screening. Gastroenterology. 2019;156(6):S-602–S-3. 10.1016/S0016-5085(19)38402-1.

[45] Daghestani A, Walker E, Mlinarevich N, Kneedler B, Berger BM. Mo1642: Diagnostic colonoscopy compliance following a positivemulti-target stool DNA test in a colorectal cancer screening-resistant population. Gastroenterology. 2018;154(6):S–780. 10.1016/S0016-5085(18)32693-3.

[46] Sweetser S, Smyrk TC, Sinicrope FA. Serrated colon polyps as precursors to colorectal cancer. Clin Gastroenterol Hepatol. 2013;11(7):760–7; quiz e54–5. 10.1016/j.cgh.2012.12.004. 23267866 PMC3628288

[47] Ahlquist DA, Zou H, Domanico M, Mahoney DW, Yab TC, Taylor WR, Next-generation stool DNA test accurately detects colorectal cancer and large adenomas. Gastroenterology. 2012;142(2):248–56; quiz e25–6. 10.1053/j.gastro.2011.10.031. 22062357 PMC4017869

[48.••] Johnson DH, Kisiel JB, Burger KN, Mahoney DW, Devens ME, Ahlquist DA, Multitarget stool DNA test: clinical performance and impact on yield and quality of colonoscopy for colorectal cancer screening. Gastrointest Endosc. 2017;85(3):657–65.e1. 10.1016/j.gie.2016.11.012. 27884518 PMC10653981

[49] Ebner D, Eckmann J, Burger KN, Mahoney DW, Devens ME, Lowrie KL, Multi-target stool DNA testing enriches detection of colorectal neoplasia by colonoscopy but yield is influenced by baseline polyp detection rates. Gastrointest Endosc. 2019;89(6):AB149–AB50. 10.1016/j.gie.2019.03.060.

[50] Eckmann JD, Kisiel JB. Response to “Colorectal cancer screening by stool DNA testing and patient emotional health”. Am J Gastroenterol. 2019;114(5):829–30. 10.14309/ajg.0000000000000218. 30950841

[51.•] Cooper GS, Markowitz SD, Chen Z, Tuck M, Willis JE, Berger BM, Evaluation of patients with an apparent false positive stoolDNA test: the role of repeat stool DNA testing. Dig Dis Sci. 2018;63(6):1449–53. 10.1007/s10620-018-5001-z. 29516325 PMC5960589

[52.•] Cotter TG, Burger KN, Devens ME, Simonson JA, Lowrie KL, Heigh RI, Long-term follow-up of patients having false-positive multitarget stool DNA tests after negative screening colonoscopy: the longhaul cohort study. Cancer Epidemiol Biomarkers Prev. 2017;26(4):614–21. 10.1158/1055-9965.Epi-16-0800. 27999144 PMC5380543

[53.•] Berger BM, Kisiel JB, Imperiale TF, Geenen DJ, Heigh RI, Mahoney DW, Low incidence of aerodigestive cancers in patients with negative results from colonoscopies, regardless of findings from multitarget stool DNA tests. Clin Gastroenterol Hepatol. 2019. 10.1016/j.cgh.2019.07.057. PMC10964931 31394289

[54] Limburg P, Mahoney D, Ahlquist D, Allawi H, Johnson S, Kaiser M, Multi-target DNA aberrations in sporadic colorectal cancer tissues do not differ between younger and older patients: 273. Am J Gastroenterol. 2019;114:S160. 10.14309/01.ajg.0000590624.23097.83.

[55] Eckmann JD, Ebner D, Bering J, Kahn A, Rodriguez EA, Devens M, Multi-target stool DNA testing in patients at increased risk for colorectal neoplasia shows similar positive predictive value to average risk patients: 290. Am J Gastroenterol. 2018;113:S162.

[56.•] Klepp P, Kisiel JB, Smastuen MC, Roseth A, Andersen SN, Vatn MH, Multi-target stool DNA test in the surveillance of inflammatory bowel disease: a crosssectional cohort study. Scand J Gastroenterol. 2018;53(3):273–8. 10.1080/00365521.2018.1424935. 29313389

[57] Eckmann JD, Ebner D, Bering J, Kahn A, Rodriguez EA, Mahoney DW, Tu1015: multi-target stool Dna testing: yield as a function of time since last colonoscopy. Gastroenterology. 2019;156(6):S-947–S-8. 10.1016/S0016-5085(19)39328-X.

